# Social network correlates of free and purchased insecticide-treated bed nets in rural Uganda

**DOI:** 10.1186/s12936-022-04347-8

**Published:** 2022-11-24

**Authors:** Sae Takada, Paul J. Krezanoski, Viola Nyakato, Vincent Bátwala, A. James O’Malley, Jessica M. Perkins, Alexander C. Tsai, David R. Bangsberg, Nicholas A. Christakis, Akihiro Nishi

**Affiliations:** 1 Division of General Internal Medicine and Health Services Research, Department of Medicine, UCLA, Los Angeles, CA USA; 2 Department of Medicine, University of California, San Francisco, San Francisco, CA USA; 3 Mbarara University of Science & Technology, Mbarara, Uganda; 4 The Dartmouth Institute for Health Policy, Clinical Practice and the Department of Biomedical Data Science, Geisel School of Medicine, Lebanon, NH USA; 5 Department of Human and Organizational Development Peabody College, Vanderbilt University, Nashville, TN USA; 6 Vanderbilt Institute of Global Health, Vanderbilt University Medical Center, Nashville, TN USA; 7 Harvard Medical School, Boston, MA USA; 8 Center for Global Health and Mongan Institute, Massachusetts General Hospital, Boston, MA USA; 9 Oregon Health & Science University-Portland State University School of Public Health, Portland, OR USA; 10 Department of Sociology, Yale Institute for Network Science, New Haven, CT USA; 11 Department of Epidemiology, Fielding School of Public Health, UCLA, Los Angeles, CA USA

**Keywords:** Malaria, Bed net, Social networks, Uganda, Insecticide-treated bed net (ITN)

## Abstract

**Background:**

Malaria is a major cause of mortality and morbidity in Uganda. Despite Uganda’s efforts to distribute bed nets, only half of households have achieved the World Health Organization (WHO) Universal Coverage Criteria (one bed net for every two household members). The role of peer influence on bed net ownership remains underexplored. Data on the complete social network of households were collected in a rural parish in southwestern Uganda to estimate the association between household bed net ownership and peer household bed net ownership.

**Methods:**

Data on household sociodemographics, bed net ownership, and social networks were collected from all households across one parish in southwestern Uganda. Bed nets were categorized as either purchased or free. Purchased and free bed net ownership ratios were calculated based on the WHO Universal Coverage Criteria. Using network name generators and complete census of parish residents, the complete social network of households in the parish was generated. Linear regression models that account for network autocorrelation were fitted to estimate the association between households’ bed net ownership ratios and bed net ownership ratios of network peer households, adjusting for sociodemographics and network centrality.

**Results:**

One thousand seven hundred forty-seven respondents were interviewed, accounting for 716 households. The median number of peer households to which a household was directly connected was 7. Eighty-six percent of households owned at least one bed net, and 41% of households met the WHO Universal Coverage Criterion. The median bed net ownership ratios were 0.67 for all bed nets, 0.33 for free bed nets, and 0.20 for purchased bed nets. In adjusted multivariable models, purchased bed net ownership ratio was associated with average household wealth among peer households (b = 0.06, 95% CI 0.03, 0.10), but not associated with average purchased bed net ownership ratio of peer households. Free bed net ownership ratio was associated with the number of children under 5 (b = 0.08, 95% CI 0.05, 0.10) and average free bed net ownership ratios of peer households (b = 0.66, 95% CI 0.46, 0.85).

**Conclusions:**

Household bed net ownership was associated with bed net ownership of peer households for free bed nets, but not for purchased bed nets. The findings suggest that public health interventions may consider leveraging social networks as tools for dissemination, particularly for bed nets that are provided free of charge.

## Background

Malaria is a major cause of mortality and morbidity in Uganda [1]. As part of the Malaria Reduction Strategic Plan, the Uganda Ministry of Health proposed universal coverage of insecticide-treated bed nets (ITNs) [1], which are effective for prevention of malaria and other mosquito-borne diseases [2, 3]. Since 2007, the World Health Organization (WHO) has called for universal coverage of bed nets for all individuals at risk of malaria, defined as owning one bed net for two people [4]. Despite efforts to distribute free ITNs through mass distribution campaigns and programmes targeting pregnant women and children [1], only 54% of households in Uganda have achieved universal coverage [5–11]. Understanding factors that predict household bed net ownership is important for improving programmes intended to achieve universal coverage.

Several studies have found associations between bed net ownership and household sociodemographic status [12, 13] and between bed net ownership and knowledge about malaria transmission [14]. The effect of social influence on bed net ownership, however, remains underexplored. A study in Ghana found an association between bed net ownership among pregnant women and the women’s perception of bed net use by people that they turn to for pregnancy advice [15]. Similarly, previous research in Uganda found that people who thought that sleeping under bed nets was normative in their villages were themselves more likely to sleep under a bed net [16]. Another study examined the extent to which the decision to adopt bed nets has spillover effects between households in sub-Saharan Africa [17]. However, this study did not measure direct interactions between households.

Social networks are understudied as potential drivers of bed net ownership. Social networks represent the set of social relationships between individuals (or households) [18]. Prior research has found that social network characteristics representing macro network structure, individual network position, and network composition influence the adoption of technology [19–22] and affect the delivery of public health interventions, particularly in resource-poor settings with limited access to information [23–25]. No studies, however, have assessed the extent to which bed net ownership among households within a social network are associated with each other. Complete social networks, also known as sociocentric networks, represent ties between all pairs of actors (in this case households) within a bounded community [26]. Studies rarely collect sociocentric network data due to the expense involved in collecting complete relational information [23].

To address these gaps identified in the literature, we capture information about the complete social network of households in a rural parish in southwestern Uganda and their bed net ownership. This unique data structure allows us to estimate the association between household bed net ownership and peer household bed net ownership. Free and purchased bed nets were analysed separately given discussion among policymakers about whether uptake of bed nets would be higher if they were distributed for free or sold for a cost [27, 28].

## Methods

### Study setting and population

This population-based study was conducted between 2011 and 2012 in a rural parish in Rwampara District, southwestern Uganda, as one of several studies in this population examining the association between social networks and health [29, 30]. In 2012, the GDP of Uganda was $790 per capita [31], with an estimated 35% of the population living on less than 2 US Dollars a day [32]. The local economy is driven primarily by subsistence agriculture, animal husbandry, and small-scale trading; food and water insecurity are common [33–35]. Nyakabare Parish was chosen among several candidate parishes given its history of low migration, long period of settlement, and clear governmental and geographic boundaries that facilitated the definition of parish boundaries. The clear parish boundaries made it suitable for assessing a sociocentric networks bounded by this geographic demarcation. The study targeted all adults in this parish who were 18 years or older during the data collection period and who considered themselves to be permanent residents of the parish. Anyone who fit those criteria and who could communicate were eligible for this study. Individuals who were too ill or otherwise unable to consent to participate were excluded from the study. Prior to data collection, community engagement meetings were conducted with local leaders and the larger community to provide parish residents with information about study activities and to elicit feedback [36].

### Data collection

Interview materials were translated and back-translated between English and Runyankore by trained research assistants, and pilot-tested to ensure cultural sensitivity and appropriateness to the local context. Data were collected in two stages. During the first stage, the research team went from household to household to conduct a parish-wide census of all eligible adults, assign them to a specific household, and obtain sociodemographic information. A household was defined as a group of people who resided together and had meals together for at least three of the past 12 months. This census was continuously updated during the data collection period. During the second stage, the research team conducted confidential, one-on-one, interview-based surveys after obtaining consent from an eligible participant. Interviews included asking about each respondent’s bed net ownership within their household as well as each respondent’s ties to other adults permanently residing within the parish.

Five name-generator questions were used to elicit from each participant with whom they had particular kinds of interactions in the past 12 months: people with whom they (1) spent free time, (2) discussed financial issues, (3) discussed health issues, (4) turned to for emotional support, (5) exchanged food (see Appendix). For each question, the participant was asked to name up to six adults residing within the parish. Names could be repeated across the name-generator questions. Given that single name-generator questions do not sufficiently capture a full social network [37], responses from the five questions were combined to represent the set of social ties among all participants.

A household-based sociocentric network was created where a tie between two households existed if a participant belonging to one household had a tie with a participant belonging to another household. A simple weighting scheme was used in which two households shared a tie with a weight of one when any member of an index household nominated any member of the other household. Households with whom the index household shared a tie are termed, “peer households.” For sensitivity analyses, a sociocentric network of household heads based only on ties between household heads was created. For this study, household head was defined as the oldest woman of reproductive age (15 to 49 years). If such a person was not available in the household, the oldest male age 15 to 49 was selected, followed by the oldest female and the oldest male [38].

### Primary variables of interest

#### Purchased and free bed net ownership

The dependent variables of interest were free and purchased bed nets owned by the household according to the survey response of the household head. The total number of bed nets owned by the household were collected, and for up to four bed nets, the locations where the bed nets were obtained were collected. Bed nets obtained from the pharmacy, market, and shops were defined as purchased, and cost approximately 15,000 Ugandan shillings (~ $6 US dollars) at the time the study was conducted. Bed nets obtained from the hospital, church, local government, non-governmental organization, and relatives were defined as free.

In accordance with WHO Universal Coverage Criterion [39], the purchased bed net ownership ratio was calculated as the number of bed nets purchased by the household over the number of members in the household divided by 2 and rounded up:

$$purchased\;bednet\;ownership\;ratio = \frac{number\;of\;nets\;purchased\;by\;household}{{rounded \left( {number\;of\;members\;in\;household/2} \right)}}.$$

For example, for a household with five members that owns two purchased bed nets, the purchased net ownership ratio would be 2/rounded (5/2) = 2/3 = 0.67. The same value of 0.67 is also obtained if the household had six members reflecting the assumption inherent in the bed net calculation that at most two persons could use the same bed net. The ratio ranges from zero to one, with higher numbers indicating more complete coverage of nets in the household. All ratios over one were converted to one, in order to not differentiate between households that have met the coverage criteria and those that have exceeded it. A household’s free bed net ownership ratio was calculated using the same equation.

#### Purchased and free bed net ownership of peer households

The primary independent variables were purchased and free bed net ownership ratios among peer households. The average purchased bed net ownership ratio among peer households was calculated as the sum of all purchased bed nets across all peer households divided by the total number of members across those households. The average free bed net ownership ratio among peer households was calculated as the sum of all free bed nets across all peer households divided by the total number of members across those households.

### Other explanatory variables

#### Household head variables

Age, sex, educational attainment, and household wealth were collected for each household head [38]. Higher socioeconomic status has been associated with bed net ownership and use [7, 40–42], and sex has been associated with both bed nets use [42] and susceptibility to peer influence [43]. Educational attainment was treated as a dichotomous variable representing whether or not the household head had completed primary school.

#### Household variables

The number of household members, the number of children under age 5, and the number of pregnant women in each household were collected. Malaria prevention programs target children under 5 and pregnant women given high morbidity and mortality among them, and their access to such interventions is an important indicator of malaria prevention efforts [1]. Prior studies have found a correlation between bed net ownership or use with presence of pregnant women [7, 41, 42] and children under 5 years [8, 41, 42, 44]) in the household. Because there was no more than one pregnant woman in any given household, a dichotomous variable was created indicating the presence of a pregnant woman in the household. Household wealth was measured using an asset index based on 26 different household items and housing characteristics based on the household heads’ responses [45]. Finally, a dichotomous variable for whether the interview was conducted during rainy season was created based on the month of the interview (September to November and March to May), as ownership and use of bed net is associated with seasonal variation in malaria risk [46].

#### Network variables

Indegree network centrality was used to measure network embeddedness. Prior research has found associations between this network variable and technology adoption [22]. Indegree network centrality was defined as the number of nominations any member of the index household received by any members of other households. A nomination held a weight of one. In addition, for each household, the average household wealth among peer households was calculated. The hypothesis was that socioeconomic status of peer households would be correlated with bed net ownership of those households. For sensitivity analyses involving the household head network, we calculated these variables based only on nominations between household heads.

### Statistical analysis

First, bivariate analyses were conducted to estimate associations between purchased bed net ownership ratio and average purchased bed net ownership ratio of peer households, age, sex, and educational attainment of the household head, household wealth, presence of children under 5 and pregnant women, whether the interview was conducted during the rainy season, household network centrality, and average wealth of peer households. Then, a multivariable linear regression model was specified in which a household’s purchased bed net ownership ratio was regressed on the average purchased bed net ownership ratio of peer households, adjusting for all the other variables. Analogous bivariate and multivariable analyses were conducted where free bed net ownership ratio was the dependent variable. Sensitivity analyses included the same analyses, but used network data about household heads only.

The packages *igraph* version 2.0 and *sna* version 2.4 were used for social network analyses in R (version 3.4.1). Because households who are connected to each other may share unmeasured influences that affect their bed net ownership, a simple linear regression model could potentially overestimate the association between a household’s bed net ownership and bed net ownership of peer households. Therefore, an autoregressive model using the linear network correlation model (*lnam*) function in the *sna* package was used that modeled peer-effects between all pairs of households by taking into account the correlation of residuals between peer households [47, 48].

## Results

### Descriptive statistics

One thousand sixty-nine individuals out of 1747 eligible individuals were interviewed. In total, 716 households were included. The response rate at the individual level was 96%. Household heads were 36 years old on average, 637 (89%) were women, and 506 (71%) did not complete primary school (Table 1). Households had a median of 5 adult household members. One tenth of households had a pregnant woman and no household had more than 1 pregnant woman. Half of the households had at least one child under 5. Households had a median number of 7 ties (interquartile range [IQR] = 4 to 11) to peer households.

**Table 1:** Household demographic characteristics and bed net ownership (n = 716)

	N (%) or median (IQR)
Household head characteristics	
Age of household head	
Less than 20	69 (9%)
20–29	256 (35%)
30–39	182 (25%)
40–49	99 (13%)
50–59	43 (6%)
60–69	40 (5%)
70 and above	47 (6%)
Sex of household head	
Female	637 (89%)
Male	79 (11%)
Educational attainment of household head	
Did not complete primary school	506 (71%)
Completed primary school	210 (29%)
Household characteristics	
Number of people in household	5 (4–7)
Households with pregnant women	74 (10%)
Households with children under 5	
No children under 5	360 (51%)
One child under 5	163 (23%)
2 children under 5	130 (18%)
3 or more children under 5	54 (8%)
Household centrality	
In-degree	7 (4–11)
Interviewed during rainy season	
Yes	245 (34%)
No	417 (66%)
Number of bed nets owned by household	
0	100 (14%)
1	158 (22%)
2	199 (28%)
3	147 (21%)
4	75 (10%)
5 or more	37 (5%)
Sources of bed nets	
Purchased	
Pharmacy	18 (1%)
Market	221 (13%)
Shop	455 (26%)
Free	
Hospital	42 (2%)
Church	3 (0%)
Local Government	743 (43%)
NGO	68 (4%)
Relatives	30 (2%)
Other/unknown	155 (9%)
Bed net ownership ratio	
All bed nets	0.67 (0.33–1.00)
Free bed nets	0.33 (0–0.67)
Purchased bed nets	0.20 (0–0.50)

One third of the households were interviewed during rainy season. Based on household head responses, 616 (86%) households owned at least one bed net, and 297 households (41%) met the WHO Universal Coverage Criterion of one bed net peer two household members. Among these 297 households, 84 (12%) met the criterion exclusively with free bed nets, and 80 (11%) met it exclusively with purchased bed nets. Forty-three percent of all bed nets were obtained from the local government, followed by 26% from shops and 13% from markets. The median bed net ownership ratio was 0.67 for all bed nets, 0.33 for free bed nets, and 0.20 for purchased bed nets.

### Purchased bed net ownership ratio

Estimates from bivariate analyses indicated that household purchased bed net ownership ratio was associated with household head age, sex, and educational attainment, household wealth, presence of a pregnant woman in the household, household head being interviewed during rainy season, household network indegree centrality, average purchased bed net ownership ratio across peer households, and average household wealth of peer households. Estimates from the multivariable model indicated that greater household wealth and greater average peer household wealth were both associated with a higher purchased bed net ownership ratio (Table 2). Average peer bed net ownership ratio was not associated with household purchased bed net ownership ratio.

**Table 2:** Correlates of household bed net ownership ratio for purchased and free bed nets in the social network of households in southwestern Uganda (N of households = 716)

Independent variables	Dependent variables			
	Purchased bed net ownership ratio		Free bed net ownership ratio	
	Bivariate analyses	Multivariable model	Bivariate analyses	Multivariable model
Household head variables				
Age	0.005 (0.004, 0.006)***	− 0.001 (− 0.002, 0.001)	0.005 (0.003, 0.006)***	− 0.001 (− 0.002, 0.001)
Sex				
Female (ref)				
Male	0.179 (0.090, 0.268)***	0.045 (− 0.010, 0.100)	0.052 (− 0.044, 0.147)	− 0.057 (− 0.111, − 0.003)*
Educational attainment				
Less than primary school (ref)				
Completed primary school	0.183 (0.122, 0.245)***	0.059 (− 0.004, 0.123)	0.045 (− 0.021, 0.111)	− 0.026 (− 0.087, 0.035)
Household variables				
Asset index	0.096 (0.086, 0.106)***	0.060 (0.037, 0.083) ***	0.087 (0.070, 0.0105)***	− 0.007 (− 0.032, 0.018)
Presence of pregnant woman	0.102 (0.010, 0.194)*	0.035 (− 0.106, 0.175)	− 0.014 (0.537, − 0.565)	− 0.139 (− 0.282, 0.004)
Number of children under 5	0.011 (− 0.015, 0.036)	− 0.044 (− 0.069, − 0.019) ***	0.134 (0.108, 0.161)***	0.078 (0.053, 0.103) ***
Interview during rainy season	0.190 (0.131, 0.248)***	0.018 (− 0.041, 0.077)	0.274 (0.210, 0.338)***	0.048 (− 0.002, 0.099)
Network variables				
Network centrality	0.009 (0.004, 0.014)***	0.005 (0.001, 0.009) *	0.003 (− 0.001, 0.008)	0.002 (− 0.002, 0.006)
Average asset index of peer households	0.111 (0.100, 0.121)***	0.066 (0.031, 0.101) ***	0.095 (0.085, 0.106)***	0.027 (− 0.009, 0.064)
Average purchased bed net ownership ratio of peer households	0.693 (0.489, 0.897)***	− 0.045 (− 0.284, 0.194)		
Average free bed net ownership ratio of peer households			0.907 (0.873, 0.942) ***	0.657 (0.464, 0.851) ***
Intercept		0.011 (− 0.013, 0.035)		− 0.072 (− 0.107, − 0.038) ***

^*^p < 0.05; **p < 0.01; ***p < 0.001

### Free bed net ownership ratio

Estimates from bivariate analyses indicated that free bed net ownership ratio was associated with household head age, household wealth, number of children under 5 in the household, household head being interviewed during the rainy season, average free bed net ownership ratio across peer households, and average household wealth of peer households. Estimates from the multivariable model indicated that male sex of household head was associated with lower free bed net ownership ratio, while a greater number of children under 5 and higher average free bed net ownership ratios of peer households were associated with higher free bed net ownership ratios (Table 2).

### Sensitivity analyses

Sensitivity analyses using network data based only on ties between household heads similarly indicated that greater household wealth and higher average household wealth of peer households were associated with higher purchased bed net ownership ratio peer estimates from the multivariable model. Likewise, the number of children under 5, being interviewed during rainy season, and average free bed net ownership ratios of peer households were associated with higher free bed net ownership ratios (Table 3).

**Table 3:** Correlates of household bed net ownership ratio for purchased and free bed nets in the social network of households heads in southwestern Uganda (N of households = 716)

Independent variables	Dependent variables			
	Purchased bed net ownership ratio		Free bed net ownership ratio	
	Bivariate analyses	Multivariable model	Bivariate analyses	Multivariable model
Household head variables				
Age	0.007 (0.006, 0.009)***	− 0.001 (− 0.002, 0.001)	0.007 (0.006, 0.009)***	− 0.001 (− 0.002, 0.001)
Sex				
Female (ref)				
Male	0.229 (0.170, 0.288)***	0.057 (0.003, 0.111)*	0.067 (0.005, 0.128)*	− 0.042 (− 0.093, 0.009)
Educational attainment				
Less than primary school (ref)				
Completed primary school	0.289 (0.225, 0.353)***	0.062 (− 0.002, 0.125)	0.057 (− 0.008, 0.123)	− 0.021 (− 0.081, 0.038)
Household variables				
Asset index	0.127 (0.117, 0.136)***	0.071 (0.050, 0.093)***	0.086 (0.068, 0.105)***	− 0.007 (− 0.029, 0.016)
Presence of pregnant woman	0.107 (− 0.059, 0.273)	0.049 (− 0.092, 0.190)	0.005 (− 0.158, 0.168)	− 0.132 (− 0.270, 0.007)
Number of children under 5	0.020 (− 0.009, 0.049)	− 0.043(− 0.068, − 0.019)***	0.131 (0.102, 0.159)***	0.067 (0.042, 0.092)***
Interview during rainy season	0.218 (0.152, 0.283)***	0.026 (− 0.033, 0.084)	0.308 (0.247, 0.369)***	0.053 (0.007, 0.100)*
Network variables				
Network centrality	0.035 (0.025, 0.045)***	0.009 (0.001, 0.017)*	0.014 (0.006, 0.022)**	0.006 (− 0.001, 0.013)
Average asset index of peer households	0.128 (0.119, 0.138)***	0.053 (0.023, 0.083)***	0.117 (0.106, 0.129)***	0.015 (− 0.017, 0.046)
Average purchased bed net ownership ratio of peer households	0.712 (0.508, 0.916)***	− 0.075 (− 0.253, 0.102)		
Average free bed net ownership ratio of peer households			0.897 (0.864, 0.931)***	0.712 (0.562, 0.863)***
Intercept		0.015 (− 0.021, 0.051)		− 0.150 (− 0.193, − 0.107)***

*p < 0.05; **p < 0.01; ***p < 0.001

## Discussion

In this study of a sociocentric network of 716 households in rural Uganda, household bed net ownership was associated with free but not purchased bed net ownership in peer households. In contrast, household wealth and educational attainment of the household head were associated with purchased bed net ownership, but not with free bed net ownership. Together, these findings suggest that mechanisms that govern the propagation of bed nets may differ based on the source of the bed nets. These findings imply that malaria control programmes may need to use a mix of free- and market-based distribution strategies to achieve universal bed net coverage [49].

This study showed that, while 86% of households owned at least one bed net, only 41% owned one bed net per two household members to meet the WHO Universal Coverage Criterion [39]. This gap between owning any bed nets and meeting the coverage criterion is similar to findings from the 2018 to 2019 Uganda Malaria Indicator Survey which found that 83% of households owned at least one bed net but only 54% met the universal coverage criterion (6), and findings from other malaria-endemic countries [9, 10].

Using the universal coverage criterion, the number of free bed nets owned per two household members was correlated with the number of free bed nets owned per two household members in peer households. An increase in the average free bed net ownership ratio from 0 (no free bed nets) to 1 (1 free bed net per two people) among peer households was associated with an increase of 0.29 in the free bed net ownership ratio for an index household. This association was not seen for ownership of purchased bed nets. Cost of a bed net is a barrier to bed net ownership in under-resourced settings [27, 28]. These findings suggest that obtaining free bed nets may be a collective activity enhanced by peer associations and mediated through women heads of households, whereas purchasing bed nets may be an individualistic activity. The findings also reinforce the potential of utilizing social networks to increase adoption of public health interventions [50], but perhaps only for free interventions. Purchased bed nets, either in addition to those available through free distribution campaigns or because households lack access to distribution campaigns and still desire malaria protection, may propagate through different mechanisms than do bed nets obtained for free.

Prior studies have found associations between bed net ownership and household wealth [9], with socioeconomic inequities in bed net ownership attenuated but still persistent following free bed net distribution campaigns [11, 51]. This study found that household wealth and household head educational attainment was associated with ownership of purchased bed nets, but not with free bed nets. These findings suggest that access to free bed nets could reduce the equity gap in bed net ownership [8]. In addition, households with a higher number of children under 5 had a higher rate of free bed net ownership, suggesting that these households may have greater access through free distribution campaigns [1]. This association was not seen for pregnant women who are also intended to receive bed nets during antenatal care visits, though there was limited power to detect this association.

Interpretation of the findings is subject to limitations. First, the data are self-reported and therefore subject to the limitations inherent to all studies based on self-report data, such as social desirability bias [52]. Second, the study was conducted in a rural community in southwestern Uganda so the results may not be generalizable to other populations or settings. However, this community may be similar to many malaria-endemic settings in sub-Saharan Africa, where the local economy features agrarian and trading enterprise, infrastructure such as piped water and electricity is rare, and household food and water insecurity are common [30, 34, 35, 53]. Third, the data are cross-sectional, limiting the ability to make causal inference. While the network autocorrelation model [47] accounts for exogenous, unmeasured influences that affect people who are connected to each other, the associational findings cannot distinguish between peer influence vs. homophily [54]. Finally, name generator questions to elicit social ties can be interpreted differently by different respondents [55], but the questions were formulated to be culturally specific with explicitly defined situations to reduce variability in interpretation [56].

## Conclusions

This cross-sectional, population-based sociocentric social network study conducted in rural Uganda found that households directly connected to other households who owned free bed nets were themselves more likely to own free bed nets. However, this peer-based association was not found for purchased bed nets. The findings suggest that public health interventions should consider utilizing social networks as tools for dissemination, particularly for interventions that are free.

## Data Availability

The datasets analysed during the current study are available from the corresponding author on reasonable request.

## Name generator questions

1. Over the last 12 months, with whom in this parish have you usually spend time for your enjoyment, relaxation, at parties, attending trainings together of your choice, watching sports games, taking alcohol together, weaving mats, or whenever you have made time for yourself (free time)? Remember, you may name from zero up to six people, and they must be at least 18 years old. Also, please remember that you may tell me names of people that you have already mentioned in response to previous questions as well.
2. Over the last 12 months, with whom in this parish have you usually talked about any kind of financial issues? This may include conversations about school feels, employment, giving, receiving, or paying loans, starting businesses, financing for big events, or other issues. Remember, you may name from zero up to six people, and they must be at least 18 years old. Also, please remember that you may tell me names of people that you have already mentioned in response to previous questions as well.
3. Over the last 12 months, with whom in this parish have you usually talked about any kind of health issues? This may include topics like your child’s health, family planning, nutrition, HIV, mental health, immunizations, sanitation methods, alcohol abuse or other issues. Remember, you may name from zero up to six people, and they must be at least 18 years old. Also, please remember that you may tell me names of people that you have already mentioned in response to previous questions as well.
4. Over the last 12 months, whom in this parish have you gone to for emotional support? This may include talking about both positive and negative topics such as deaths, marriages, births, loss of job, or other topics of emotional importance to you. Remember, you may name from zero up to six people, and they must be at least 18 years old. Also, please remember that you may tell me names of people that you have already mentioned in response to previous questions as well.
5. Over the last 12 months, with whom have you shared, borrowed, received, or exchanged food Please name only people who stay outside of your household, but in your parish. Remember, you may name from zero up to six people, and they must be at least 18 years old. Also, please remember that you may tell me names of people that you have already mentioned in response to previous questions as well.

## Abbreviations

ITN: Insecticide-treated bed net
WHO: World Health Organization
